# Analyzing and minimizing PCR amplification bias in Illumina sequencing libraries

**DOI:** 10.1186/gb-2011-12-2-r18

**Published:** 2011-02-21

**Authors:** Daniel Aird, Michael G Ross, Wei-Sheng Chen, Maxwell Danielsson, Timothy Fennell, Carsten Russ, David B Jaffe, Chad Nusbaum, Andreas Gnirke

**Affiliations:** 1 Genome Sequencing and Analysis Program, Broad Institute of MIT and Harvard, 320 Charles Street, Cambridge, MA 02141, USA; 2 Learning Community C, Cambridge Rindge and Latin School, 459 Broadway, Cambridge, MA 02138, USA; 3 Genome Sequencing Platform, Broad Institute of MIT and Harvard, 320 Charles Street, Cambridge, MA 02141, USA

## Abstract

Despite the ever-increasing output of Illumina sequencing data, loci with extreme base compositions are often under-represented or absent. To evaluate sources of base-composition bias, we traced genomic sequences ranging from 6% to 90% GC through the process by quantitative PCR. We identified PCR during library preparation as a principal source of bias and optimized the conditions. Our improved protocol significantly reduces amplification bias and minimizes the previously severe effects of PCR instrument and temperature ramp rate.

## Background

The Illumina sequencing platform [1], like other massively parallel sequencing platforms [2,3], continues to produce ever-increasing amounts of data, yet suffers from under-representation and reduced quality at loci with extreme base compositions that are recalcitrant to the technology [1,4-6]. Uneven coverage due to base composition necessitates sequencing to excessively high mean coverage for *de novo *genome assembly [7] and for sensitive polymorphism discovery [8,9]. Although loci with extreme base composition constitute only a small fraction of the human genome, they include biologically and medically relevant re-sequencing targets. For example, 104 of the first 136 coding bases of the retinoblastoma tumor suppressor gene *RB1 *are G or C.

Traditional Sanger sequencing has long been known to suffer from problems related to the base composition of sequencing templates. GC-rich stretches led to compression artifacts. Polymerase slippage in poly(A) runs and AT dinucleotide repeats caused mixed sequencing ladders and poor read quality. Processes upstream of the actual sequencing, such as cloning, introduced bias against inverted repeats, extreme base-compositions or genes not tolerated by the bacterial cloning host. Gaps due to unclonable sequences had to be recovered and finished by PCR [10], or, in some cases, by resorting to alternative hosts [11]. Cloning bias hindered efforts to sequence the AT-rich genomes of *Dictyostelium *[12] and *Plasmodium *[13] and excluded the GC-rich first exons of about 10% of protein-coding genes in the dog (K Lindblad-Toh, personal communication) from an otherwise high-quality reference genome assembly [14].

New genome sequencing technologies [1-3,15-17] no longer rely on cloning in a microbial host. Instead of ligating DNA fragments to cloning vectors, the three major platforms currently on the market (454, Illumina and SOLiD) involve ligation of DNA fragments to special adapters for clonal amplification *in vitro *rather than *in vivo*. Due to the massively parallel nature of the process, standardized reaction conditions must be applied to amplify and sequence complex libraries of fragments that comprise a wide spectrum of sequence compositions. All three platforms display systematic biases and unevenness as the observed coverage distributions are significantly wider than the Poisson distribution expected from unbiased, random sampling [18].

The Illumina sequencing process consists of i) library preparation on the lab bench, ii) cluster amplification, sequencing-by-synthesis and image analysis on proprietary instruments, followed by iii) post-sequencing data processing. Bias can be introduced at all three stages. For example, high cluster densities on the Illumina flowcell suppress GC-rich reads. Changes to sequencing kits, protocols and instrument firmware can affect the base composition of sequencing data. Moreover, bias is known to vary between laboratories, from run to run or even from lane to lane on the same flowcell. Such variability and instability in the system confound comparative studies [19,20] and render systematic bias investigations difficult.

Here, we set out to evaluate sources of bias during Illumina library preparation and to ameliorate the effects. We undertook a systematic dissection of the process, using quantitative PCR (qPCR) instead of Illumina sequencing as a quick and system-independent read-out for base-composition bias. We identified library amplification by PCR as by far the most discriminatory step. We examined hidden factors such as make and model of thermocyclers and modified the thermocycling protocol. We tested alternative PCR enzymes and chemical ingredients in amplification reactions. Finally, we validated the qPCR results by Illumina sequencing. Our optimized protocol amplifies sequencing libraries more evenly than the standard protocol and minimizes the previously severe effects of PCR instrument and temperature ramp rate.

## Results

### Following a diverse panel of loci through the Illumina library preparation

The Illumina library preparation protocol is a multi-step process consisting of shearing of the input DNA, enzymatic end repair, 5'-phosphorylation and 3'-single-dA extension of the resulting fragments, adapter ligation, size fractionation on an agarose gel and PCR amplification of adapter-ligated fragments. Bias can potentially be introduced at any step, including the physical clean-up steps that remove proteins, nucleotides and small DNA fragments.

Since virtually all genomes have their base composition in a narrow %GC range, we used a composite genomic DNA sample with a range of base composition spanning almost the entire spectrum as a test substrate throughout our investigation of sources of bias. We started with an equimolar mixture of DNA prepared from *Plasmodium falciparum *(genome size 23 Mb; GC content 19%), *Escherichia coli *(4.6 Mb; 51% GC) and *Rhodobacter sphaeroides *(4.6 Mb; 69% GC). The composite 32-Mb 'PER' genome is about 100 times smaller than a typical mammalian genome, making it a more tractable size for our analyses. A histogram of the %GC distribution of 50-bp windows in the three genomes is shown in Figure S1 in Additional file 1.

**Figure 1:**
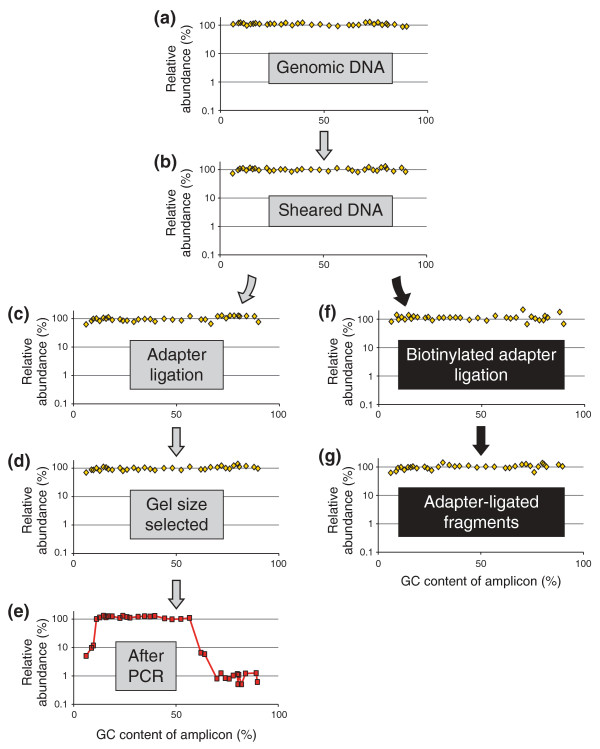
**Tracing a diverse panel of loci through the Illumina library preparation**. **(a-e) **At five steps in the standard protocol aliquots were removed and analyzed for base-composition bias by qPCR. **(f,g) **To isolate and analyze the ligation-competent population of DNA fragments, a separate ligation reaction with biotinylated adapters was performed followed by streptavidin capture of fragments carrying at least one adapter. The quantity of each amplicon in a given sample was divided by the mean quantity of the two amplicons closest to 50% GC. The resulting relative abundances of amplicons were plotted on a log_10 _scale over their respective GC contents.

We next developed a panel of qPCR assays that define amplicons ranging from 6% to 90% GC (Table S1 in Additional file 2). The amplicons were very short (50 to 69 bp) and thus allowed us to perform qPCR assays on sheared 'PER' DNA and on aliquots drawn at various points along the protocol (Figure 1). We determined the abundance of each locus relative to a standard curve of input 'PER' DNA. To adjust for differences in DNA concentration, we normalized the calculated quantities relative to the average quantity of the 48% GC and 52% GC amplicons in each sample.

The input 'PER' genomic DNA is unbiased per definition. As expected, a scatter plot of the normalized quantity of each amplicon over its GC content was essentially flat from 6% to 90% GC when plotted on a log scale, validating the qPCR-based bias assay (Figure 1a). Shearing the DNA did not lead to any obvious skewing of the base composition (Figure 1b), nor did the subsequent three enzymatic reaction steps up to the adapter ligation (Figure 1c). This is not surprising since up to this point no explicit DNA-fractionation step had taken place other than the clean-up steps. Analyzing the ligation mixture of adapter-ligated fragments by qPCR would not reveal potential bias during any of the enzymatic reactions necessary for ligating the adapter to the sheared DNA fragments because the mixture presumably includes some adapter-less fragments.

To perform a bias assay exclusively on the adapter-ligated fraction, we set up a ligation with non-phosphorylated biotinylated adapters, isolated the adapter-ligated DNA fragments by streptavidin capture and released the captured insert fragments by denaturation for analysis by qPCR. We saw very little, if any, systematic GC bias in the adapter-ligated fraction (Figure 1f,g), and thus no evidence for strong discrimination based on base composition during any of the preceding enzymatic reactions and clean-up steps.

Excising a narrow size range (corresponding to approximately 170- to 190-bp genomic fragments) from a preparative agarose gel did not skew the base composition (Figure 1d). However, as few as ten PCR cycles using the enzyme formulation (Phusion HF DNA polymerase) and thermocycling conditions prescribed in the standard Illumina protocol depleted loci with a GC content > 65% to about a hundredth of the mid-GC reference loci (Figure 1e). Amplicons < 12% GC were diminished to approximately one-tenth of their pre-amplification level. Between the steep flanks on either side, the GC-bias plot was essentially flat. Its plateau phase (defined as the segment on the %GC axis with no more than one data point below a relative abundance of 0.7) ranged from 11% to 56% GC.

### Comparing three thermocyclers at their default ramp speeds

**Figure 2:**
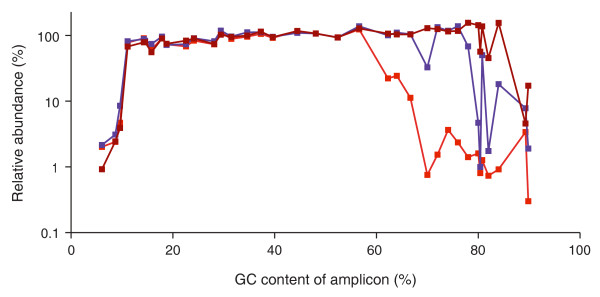
**Effect of temperature ramp rates**. The standard PCR protocol with Phusion HF DNA polymerase and short initial (30 s) and in-cycle (10 s) denaturation times was performed on three different thermocyclers at their respective default temperature ramp settings. Heating and cooling rates were 6°C/s and 4.5°C/s on thermocycler 1 (bright red line), 4°C/s and 3°C/s on thermocycler 2 (purple line) and 2.2°C/s and 2.2°C/s on thermocycler 3 (dark red line).

PCR protocols published by kit manufacturers or in the scientific literature usually specify the temperature and duration time of each thermocycling step (for example, 10 s at 98°C for the denaturation step during each cycle for the PCR enrichment of Illumina libraries) but rarely the temperature ramping speed or the make and model of the thermocycler. For the experiment shown in Figure 1 (and for a replicate experiment shown in Figure 2, bright red line), we used the default heating and cooling rates (6°C/s and 4.5°C/s, respectively) on thermocycler 1 (see Materials and methods for make and model).

Running the PCR protocol on thermocyler 2 (at its default heating and cooling rates of 4°C/s and 3°C/s, respectively) extended the plateau to 76% GC (Figure 2, purple). Thermocyler 3 had the slowest default ramp speed (2.2°C/s). Its bias plot was flat from 13% to 84% GC before dropping down to one-tenth the level for the two most GC-rich loci (Figure 2, dark red). These results are consistent with the notion that an overly steep thermoprofile does not leave sufficient time above a critical threshold temperature, causing incomplete denaturation and poor amplification of the GC-rich fraction.

### Optimizing the PCR conditions

To develop a robust protocol that produces consistent results across a wide range of ramp speeds and thermocyclers, we chose to optimize the reaction conditions on thermocycler 1, the worst performer, at its fast default ramp speed. We reasoned that a protocol that works well on this machine would also work on a slower-ramping thermocycler.

**Figure 3:**
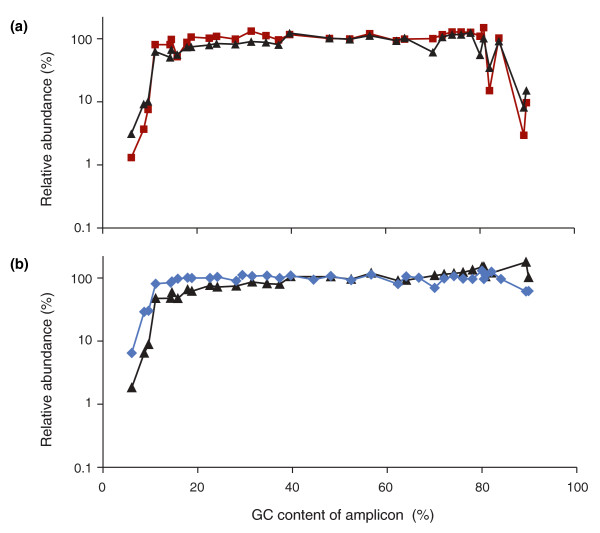
**Optimizing the PCR conditions**. **(a) **Neither extending the denaturation times (dark red squares) nor adding 2M betaine (black triangles) is sufficient to recover extremely GC-rich DNA fragments by PCR with Phusion HF. **(b) **Combining long denaturation and 2M betaine is effective for the high-GC fraction (black triangles) but the profile is not as even over the entire GC spectrum as after PCR with AccuPrime Taq HiFi (blue diamonds) using extended denaturation times and a lower temperature (65°C) for primer annealing and extension.

Simply extending the initial denaturation step (from 30 s to 3 minutes) and the denaturation step during each cycle (from 10 s to 80 s) overcame the detrimental effects of the overly fast ramp rate, albeit without fully restoring the extremely high-GC fraction (Figure 3a, dark red squares). Long denaturation produced a library of similar quality as the shorter denaturation on the slow-ramping thermocyler 3 (Figure 2, dark red).

Adding 2M betaine without changing the thermoprofile had an equivalent effect on moderately high-GC fragments but led to a slight depression of loci in the 10% to 40% GC range (Figure 3a, black triangles). Adding 2M betaine and extending the denaturation times rescued - in fact slightly over-represented - loci at the extreme high end of the GC spectrum at the expense of low-GC fragments (Figure 3b, black triangles), shifting the plateau to the right (23 to 90% GC).

By substituting Phusion HF with the AccuPrime Taq HiFi blend of DNA polymerases and fine-tuning the thermoprofile, specifically by prolonging the denaturation step and lowering the temperature for primer annealing and extension from 72°C to 65°C, we obtained the GC-bias profile shown in Figure 3b (blue diamonds). These conditions restored extremely high-GC loci almost fully while avoiding the suppression of moderately low-GC amplicons seen with Phusion HF and 2M betaine (black triangles). The plateau ranged from 11% to 84% GC with only a very slight drop above. Lowering the temperature for the extension even further (to 60°C) shifted the balance slightly in favor of AT-rich loci at the expense of GC-rich ones (see below).

We performed a side-by-side comparison of the AccuPrime Taq HiFi PCR protocol on the fastest-ramping thermocycler 1 and on the slowest-ramping thermocycler 3 and found few, if any, differences in the GC-bias curves (Figure S2a in Additional file 1). We also tested it on adapter-ligated fragment libraries that had been sheared and size-selected to approximately 360-bp instead of 180-bp inserts. The GC profiles of PCR-amplified larger-insert libraries were almost as flat as that of a small-insert control library amplified in parallel, with a slightly rounder shoulder, reaching the flat phase at 17% instead of 13% GC (Figure S2b in Additional file 1).

### Direct comparison of fragment library and sequencing reads

The qPCR assay measures the composition of the PCR-amplified library. It is likely that downstream steps such as cluster amplification, sequencing-by-synthesis, image analysis and off-instrument data processing also introduce bias. To directly compare input libraries and the final output data, that is, the quality-filtered and aligned Illumina reads, we sequenced four 400-bp fragment libraries for which we also had qPCR data and counted the sequencing reads covering the very same loci.

**Figure 4:**
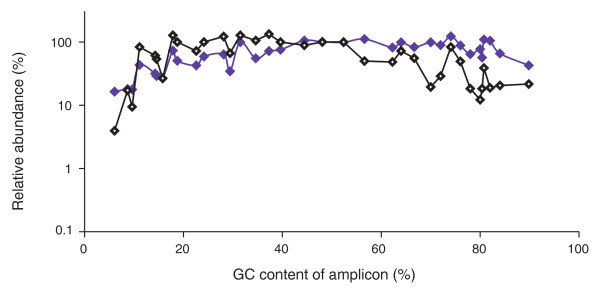
**Comparing input library and output sequencing data**. Shown is the relative abundance of loci in the library as determined by qPCR (purple) and the relative abundance of Illumina sequencing reads covering these loci in one lane of Hi-Seq data (black). Both data sets were normalized to the average of the two loci closest to 50% GC.

As shown in Figure 4, for a library amplified with AccuPrime Taq HiFi using 60°C for the primer extension step, sequencing and qPCR GC profiles closely track each other, including some of the pronounced ups and downs that may reflect amplification traits of individual loci, such as sequence context or potential for hairpin formation, not captured in their average GC content indicated on the x-axis. A superimposition of qPCR and sequencing data for three differently amplified libraries is available in Figure S3 in Additional file 1.

We noted some outliers. For example, amplicons with approximately 70% or 80% GC received less sequence coverage than their neighbors in %GC space, despite relatively high abundance in the library. Close examination of amplicons > 50% GC suggested an effect of sequence context. We found the %GC of a 250-bp window centered on the amplicons a better predictor of under-coverage than the %GC of the amplicons proper (Figure S4 in Additional file 1). The systematic drop in sequence coverage with increasing GC content was not caused by a proportionate under-representation of high-GC loci in the library, indicating that there is bias downstream of library preparation.

### Genome-wide sequence coverage

Our test loci, which had been selected in part based on their ability to be amplified by PCR, may or may not be true representatives of their respective base compositions at large. To measure sequencing bias genome-wide, we calculated the average ratio of observed to expected (unbiased) coverage for 50-bp sliding windows. Superimposing genome-wide and loci-specific bias data, each normalized relative to the mid-GC (48 to 52%) fraction, showed that the selected loci were, by and large, good proxies for their respective %GC categories - despite the distinct amplification behavior of individual loci (Figure S5 in Additional file 1).

**Figure 5:**
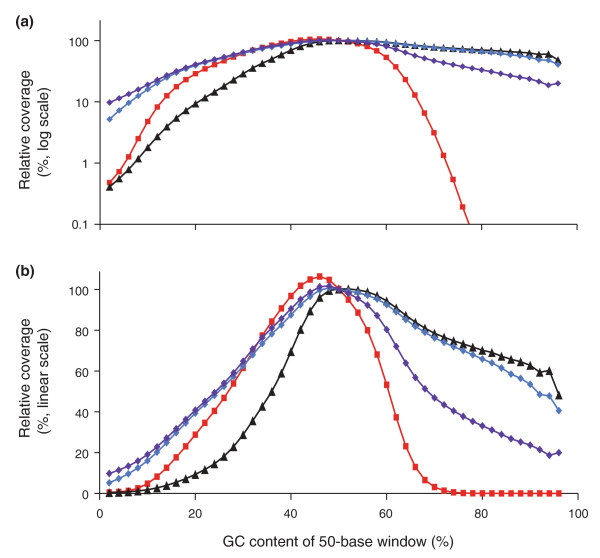
**'PER' genome-wide base composition bias curves**. **(a,b) **Shown is the GC bias in Illumina reads from a 400-bp fragment library amplified using the standard PCR protocol (Phusion HF, short denaturation) on a fast-ramping thermocycler (red squares), Phusion HF with long denaturation and 2M betaine (black triangles), AccuPrime Taq HiFi with long denaturation and primer extension at 65°C (blue diamonds) or 60°C (purple diamonds). To calculate the observed to expected (unbiased) read coverage, the number of reads aligning to 50-bp windows at a given %GC was divided by the number of 50-bp windows that fall in this %GC category. This value was then normalized relative to the average value from 48% through 52% GC and plotted on a log_10 _scale (a) or linear scale (b).

The standard Phusion HF PCR (short denaturation and fast ramp) depleted sequences > 70% GC to less than a hundredth of the mid-GC reference windows (Figure 5, red squares). Adding betaine and prolonging the denaturation step rescued the high-GC fraction efficiently and thoroughly (Figure 5, black triangles): 50-bp windows with up to 94% GC still received more than half the mean coverage of those with approximately 50% GC, demonstrating that stretches of 50 bases consisting almost entirely of Gs and Cs can be sequenced, provided they are present in the library. However, this gain of high-GC sequences came at the expense of high-AT sequences, which suffered a significant loss compared to the standard Phusion HF library.

Consistent with the qPCR data, libraries amplified with AccuPrime Taq HiFi were less skewed than libraries amplified with Phusion. Extending the annealed primer with AccuPrime Taq HiFi at 65°C (Figure 5, blue diamonds) outperformed both Phusion reactions at the low-GC end while retaining the high-GC fraction almost as well as Phusion with betaine (Figure 5, black triangles). Lowering the extension temperature to 60°C (Figure 5, purple diamonds) returned even more low-GC sequences while diminishing the yield of GC-rich reads somewhat. Extension at 60°C produced an amplified library wherein all bins of 50-bp windows between 2% and 96% GC received at least one-tenth the average coverage of the mid-GC reference.

No single PCR protocol was ideal. The best protocol for high GC, Phusion HF with betaine, led to poor representation of high-AT loci. The protocol that worked best for high AT, AccuPrime Taq HiFi with primer extension at 60°C, compromised the high-GC fraction. A pool of two differently amplified libraries would be more complex than either library alone, but would also add cost by doubling the amount of library construction required. It would still be biased and, when sequenced, produce an intermediate GC-bias profile similar to those shown in Figure S6 in Additional file 1 that were generated by pooling sequencing reads.

We also calculated the fraction of the genome that received less than one-tenth the mean genome-wide coverage (Table 1). By this measure, AccuPrime Taq HiFi PCR with primer extension at 60°C was clearly the best amplification condition for the AT-rich *P. falciparum *genome, and overall, for the composite 'PER' genome, 71% of which consists of *P. falciparum *DNA. This method was slightly worse than the 65°C extension protocol for the GC-rich *R. sphaeroides *genome, for which long-denaturation PCR with Phusion in the presence of betaine came out on top. The *E. coli *genome was very evenly covered by three conditions. Only the standard PCR protocol with Phusion HF and short denaturation, when performed with an overly fast temperature ramp, left more than 0.5% of the *E. coli *genome under-covered.

**Table 1:** Percentage of bases covered at less than one-tenth of the mean 'PER'-wide coverage

PCR condition	*P. falciparum*	*E. coli*	*R. sphaeroides*	'PER'
Phusion HF short (standard) denaturation, fast ramp	41%	0.59%	95%	42%
Phusion HF long denaturation, 2M betaine	45%	0.00011%	0.0096%	33%
AccuPrime Taq HiFi long denaturation, extension at 65°C	20%	0.00015%	0.032%	14%
AccuPrime Taq HiFi long denaturation, extension at 60°C	8.8%	0.00017%	0.085%	6.4%

### Rescuing GC-rich loci in the human genome

**Figure 6:**
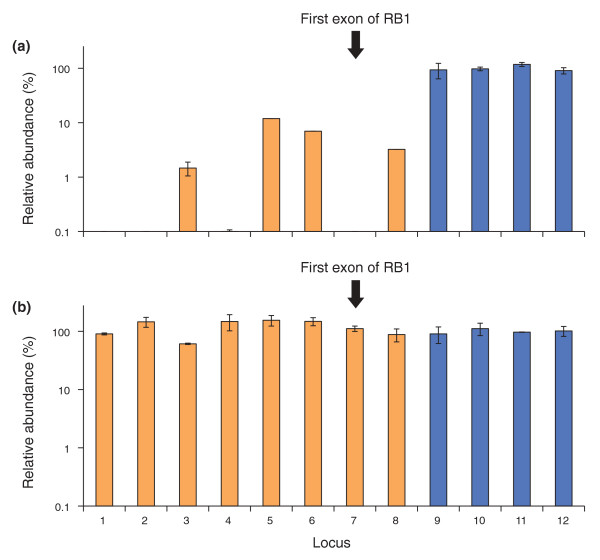
**Optimized PCR conditions rescue GC-rich promoter regions in the human genome**. **(a,b) **A 180-bp fragment library of human DNA was amplified using (a) standard conditions (Phusion HF, short denaturation) or (b) optimized conditions (AccuPrime HiFi, long denaturation, extension at 65°C) on the fast-ramping thermocycler 1. The amplified libraries were analyzed by qPCR. Orange bars indicate the quantity of eight GC-rich loci near gene promoters relative to the mean quantity of four size-matched control loci (blue bars; mean set to 100% in each graph). Error bars represent the range of two measurements averaged to calculate the quantity of each locus. Locus 7 is the first protein-coding exon of the tumor suppressor gene *RB1*.

To test if our optimized conditions improve the representation of biologically relevant loci in the human genome, we developed qPCR assays for eight GC-rich loci near gene promoters and four size-matched control loci. All eight test loci had been under-represented in previous sequencing runs with standard PCR-amplified libraries. We amplified a fragment library of human DNA on the fast-ramping thermocycler 1 using the standard Phusion and the AccuPrime Taq HiFi (extension at 65°C) protocols. The first protein-coding exon of the tumor suppressor gene *RB1 *was below the detection limit in the standard library (Figure 6a) and near unity (109% of the average of the four control loci) in the improved library (Figure 6b). The mean relative abundance of all eight test loci rose from 3% (range 0 to 11%) to 116% (range 60 to 153%).

### Comparison of PCR-amplified and PCR-free Illumina libraries

Kozarewa *et al*. [21] developed a protocol for Illumina sequencing without PCR to amplify and enrich adapter-ligated DNA fragments. We sequenced a PCR-amplified and a PCR-free human 180-bp fragment library side-by-side on an Illumina Hi-Seq flowcell and calculated the mean coverage (relative to the mean genome-wide coverage) of a larger set of GC-rich loci (Table S3 in Additional file 2). The 100 test loci were 200 bp in length, located on or near annotated transcription start sites, had a mean GC content of 80% (standard deviation 5%) and were known to be poorly covered in previous whole-genome sequencing runs. By this measure, the PCR-amplified library (AccuPrime Taq HiFi with extension at 65°C) and the PCR-free library performed equally: the mean coverage of the test loci was 28% in both data sets, a 3.6-fold under-representation.

**Figure 7:**
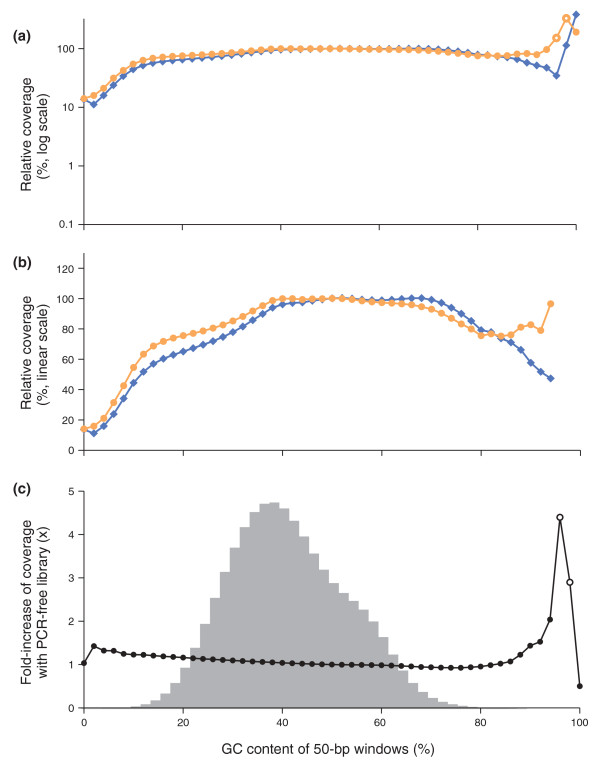
**Sequencing bias with PCR-amplified and PCR-free libraries**. **(a,b) **Shown is the mean normalized coverage of 50-bp windows in the human genome having the GC-content indicated on the x-axis for a PCR-free (orange dots) and a PCR-amplified (blue diamonds) Illumina sequencing library. Both fragment libraries had approximately 180-bp inserts. The PCR amplification was performed with AccuPrime Taq HiFi (long denat., primer extension at 65°C). The coverage was plotted on a log_10 _(a) and a linear scale (b). The data points at extremely high GC, where the reads from the PCR-free library had a mean base quality of less than Q20 (open symbols), were omitted in the middle panel (b). **(c) **The ratios of the two curves in (a,b), that is, the fold-increase in mean coverage by sequencing a PCR-free library instead of a PCR-amplified library. The shaded histogram is the %GC distribution of 50-bp windows in the human genome. More than 99.9% of all 50-bp windows in the genome contain 8% to 88% GC and received a less than 1.25-fold increase in coverage. Less than 0.01% of all 50-bp windows contain 90% or more GC. The open circles at 96% and 98% GC denote data for which the mean base quality of the reads from the PCR-free library was below Q20.

By sequencing the PCR-amplified library, 50-bp windows from 12% to 92% received at least half the mean coverage of those with 50% GC (Figure 7a,b). Only about 0.2% of 50-bp windows in the human reference genome - and less than 0.02% of 50-bp windows that overlap with the human exome - fall outside this range. With the PCR-free library, the mean relative coverage of GC-rich loci stayed near or above unity all the way to 100% GC. The PCR-free library was also slightly better for AT-rich loci, with up to 1.4-fold better coverage of 50-bp stretches containing only one G or C. From 8% to 88% GC, the fold increase by sequencing an unamplified fragment was less than 1.25 (Figure 7c). More than 99.9% of all 50-bp windows in the human genome fall in this category.

We note that skipping the PCR step during library preparation does not necessarily yield unbiased Illumina sequencing reads, presumably due to bias introduced further downstream in the sequencing process.

## Discussion

In this study, we traced a diverse panel of qPCR amplicons through the standard Illumina library construction process to define sources of bias in the Illumina sequencing process and to enable us to develop protocols that ameliorate bias. We identified the enrichment PCR step as the primary source of base-composition bias in fragment libraries and developed an optimized PCR protocol that produces libraries that are far less skewed than standard PCR-amplified Illumina libraries. We note that substantial bias is added at downstream steps on the Illumina instrument. Two of these steps, cluster amplification and sequencing-by-synthesis, also involve primer extension by DNA polymerases. Nonetheless, the benefit of a more evenly amplified fragment library carries through to the very end of the process with sequencing reads covering GC-rich and AT-rich loci that had little if any coverage before.

We found that hidden factors in the protocol, in particular the thermocycler and temperature ramp rate, can play a surprisingly big role in introducing bias. We reasoned that it would be impractical to standardize the make and model of PCR machines across the Illumina sequencing community. It would be similarly difficult to universally calibrate machine performance by adjusting the temperature ramp rates of different types of instruments. We therefore optimized the reaction conditions on the PCR machine with the fastest heating and cooling rate - the machine that performed most poorly with the standard protocol. We extended the denaturation step to provide sufficient time above the temperature threshold necessary for complete denaturation of GC-rich DNA fragments no matter how steep the thermoprofile.

Long and, presumably, complete denaturation alone does not rescue extremely GC-rich fragments in PCR reactions with Phusion HF polymerase, an enzyme with relatively weak strand-displacement activity, potentially limiting its ability to polymerize through hairpins on the template strand. Betaine may help to keep a GC-rich template single-stranded, but it may also cause premature dissociation of the newly synthesized strand from an AT-rich template.

AccuPrime Taq HiFi is a blend of *taq *polymerase, *pyrococcus *polymerase and a proprietary accessory protein added by the manufacturer to improve the priming specificity. It is conceivable that this accessory protein (which may have single-strand binding and stabilization activity) also helps to displace strands and melt hairpin structures during the polymerization. It is also possible that the excellent performance is simply due to the complementary strengths and synergy of two different enzymes working together, or to the chemical composition of the reaction buffer.

Experimental noise and imperfections in the data notwithstanding, the qPCR-based GC-bias profiles were reproducible and highly informative and predictive. Our test loci appear to be good proxies for their respective %GC bins that allow extrapolation to the genome at large - despite distinct amplification 'personalities' of individual loci.

Interestingly, PCR-induced depletion of the high-GC fraction can largely be prevented whereas under-representation of AT-rich fragments can only be slightly ameliorated at best - for example, by lowering the temperature during the primer extension. Testing additional PCR enzymes, buffers and reaction conditions will be necessary to further improve the representation of the AT-rich fraction. Ironically, it was our inability to find PCR conditions that work well for AT-rich fragments that led us to posit anti-AT bias during end-repair of adapter ligation as the root cause of the AT-deficit. However, when we examined this hypothesis directly using biotinylated adapters, we did not see significant ligation bias of a magnitude that could possibly explain the lack of AT-rich fragments in PCR-amplified fragment libraries (Figure 1).

We note that none of our conditions work equally well at rescuing, at the same time, under-representation of regions that are either extremely GC-rich or GC-poor. At the time of this writing, by our assay, PCR with AccuPrime Taq HiFi at a low primer-extension temperature is the best compromise. We did not screen and test an exhaustive list of PCR enzymes and reaction conditions. It is possible that other enzymes would perform as well or even better. Some of them may be superior in other respects, such as fidelity, size bias, cross-platform compatibility, costs or lot-to-lot variability. However, our simple and quick qPCR bias assay will enable a wider search for optimal PCR reagents and amplification conditions.

Obviously, the best way to avoid bias during PCR is to avoid library amplification by PCR altogether [6,21]. On the other hand, PCR-free libraries require relatively large amounts of input DNA and are thus impractical for many sample types. Furthermore, there is no enrichment of sequenceable fragments carrying adapters on both ends, and the yield of such fragments is very sensitive to variations in DNA quality and purity, which in turn can affect the efficiency of end repair and adapter ligation. We also note that preparing PCR-free libraries alone does not necessarily guarantee unbiased sequencing data as significant bias is introduced elsewhere in the process.

Importantly, PCR-free protocols are not readily amenable to automation and are therefore not the best choice as the default protocol in high-throughput sequencing facilities such as the Broad Institute Genome Sequencing Platform, where currently about 1,000 Illumina sequencing libraries are made per week to support a diverse portfolio of sequencing projects. Improved PCR conditions like the one described here will likely satisfy the vast majority of projects. Enhancing the coverage of high-GC loci is critical for human genome and exome sequencing in cancer and medical genetics, the major sequencing applications in terms of bases generated. Solving the loss of AT-rich loci remains a challenge, but has less of an impact on human genome sequencing and on the sequencing field as a whole. We expect that PCR-free methods, which are invaluable and superior for extreme base compositions at both ends of the %GC spectrum, will be reserved for projects that are most sensitive to base-composition bias and can supply input DNA of sufficient quality and quantity.

## Conclusions

qPCR is an inexpensive and quick assay for representational bias in Illumina fragment libraries. Our optimized PCR conditions are significantly better and more robust than the standard protocol in that they amplify more evenly across a wider range of base compositions and minimize the previously detrimental effect of fast-ramping thermocyclers. By optimizing instead of eliminating the PCR-amplification step, our protocol is easy to implement in high-throughput production and does not increase the DNA input requirements for routine Illumina library construction.

## Materials and methods

### Genomic DNA

DNA from *P. falciparum *3D7 was a gift of Dr Daniel Neafsey (Broad Institute). DNA from *E. coli *K12 MG1655 and *R. sphaeroides *2.4.1, kindly prepared by Dr Louise Williams (Broad Institute), was obtained from the Broad Institute Genomic Sequencing Sample Repository. The equimolar composite 'PER' DNA sample was a 5:1:1 mixture (by mass) of the three DNAs. The human DNA was NA12878 (Coriell Institute, Camden, NJ, USA).

### Standard Illumina fragment libraries

Illumina fragment libraries were constructed using Illumina paired-end DNA sample prep kit v1 with the following modifications. DNA (3 μg in 280 μl TE buffer) was sheared for 6 minutes on an S2 sonicator (Covaris, Woburn, MA, USA). The settings for short-insert fragment libraries were 5% duty cycle, intensity 10, and 200 cycles per burst. For long-insert fragment libraries, the intensity was reduced to 5. The modes of short and long fragment-size distributions were approximately 225 bp and 325 bp, respectively. End-repair reactions (56 μl) contained 1× T4 DNA ligase buffer (NEB, Ipswich, MA, USA), 1.4 mM ATP, 0.4 mM dNTPs, 0.1 mg/ml bovine serum albumin, 15 units T4 DNA polymerase (NEB), 50 units T4 polynucleotide kinase (NEB) and were incubated stepwise for 15 minutes at 12°C and 15 minutes at 25°C. The non-templated 3'-single-dA extension was performed for 30 minutes at 37°C in 50 μl containing 1× Klenow buffer (NEB), 0.2 mM dATP and 15 units Klenow exo^- ^(NEB). Adapter ligations (50 μl) contained 1× Quickligation buffer (NEB), 3 μl annealed paired-end adapter oligonucleotides (Illumina, San Diego, CA, USA), 5 units T4 DNA ligase (NEB) and were carried out for 15 minutes at 25°C. All reaction clean-ups were performed using a MinElute PCR purification kit (Qiagen, Hilden, Germany). Fragment libraries were size-selected on 3% NuSieve 3:1 (Lonza, Basel, Switzerland) agarose gels run in 1× TAE buffer. SYBR Green (Invitrogen, Carlsbad, CA, USA) stained DNA was visualized on a DarkReader (Clare Chemicals, Dolores, CO, USA). Gel slices were excised 90 bp larger than the desired insert size, that is, 250 to 290 bp for (180 ± 20)-bp inserts, 410 to 490 bp for (360 ± 40)-bp inserts, and 450 to 530 bp for (400 ± 40)-bp inserts. Size-selected DNA was purified with a Qiagen MinElute gel extraction kit and quantified using the Quant-iT dsDNA HS assay (Invitrogen).

### Library amplification by PCR

PCR with Illumina PE 1.0 and 2.0 enrichment primers was performed in 50-μl reactions containing 1 to 2 ng of size-selected small-insert (approximately 180 bp) fragment libraries or 2 to 4 ng of size-selected large-insert (approximately 360 bp or 400 bp) fragment libraries. Standard reactions contained 1× Phusion High-Fidelity PCR master mix with HF buffer (NEB). Standard (short denaturation) thermocycling for PCR with Phusion was 30 s at 98°C for the initial denaturation followed by 10 cycles of 10 s at 98°C, 30 s at 65°C and 30 s at 72°C and a final extension for 5 minutes at 72°C. The 'long denaturation' Phusion thermocycling protocol was 3 minutes at 98°C; 10 × (80 s at 98°C, 30 s at 65°C, 30 s at 72°C); 10 minutes at 72°C. Betaine (5 M stock solution) was from USB (Cleveland, OH, USA). One unit of AccuPrime Taq DNA polymerase High Fidelity (Invitrogen) was used in 50 μl 1× AccuPrime PCR buffer II. The thermoprofile of AccuPrime Taq HiFi reactions included the same long denaturation steps as the 'long denaturation' Phusion protocol above: 3 minutes at 98°C; 10 × (80 s at 98°C, 90 s at 65°C or 60°C); 10 minutes at 65°C or 60°C. Thermocycler 1 was a Mastercycler epgradient S (Eppendorf, Hamburg, Germany). Thermocycler 2 was an Eppendorf Mastercycler epgradient (no 'S'). Thermocycler 3 was a Gene Amp PCR System 9700 with gold-plated solid silver block (Applied Biosystems, Foster City, CA, USA) and was run in 9600 emulation mode. PCR products were purified with 1.8× AmPure XP beads (Beckman Coulter Genomics, Danvers, MA, USA) and eluted in Qiagen's EB buffer.

### PCR-free fragment libraries for Illumina sequencing

Shearing, end-repair, single-dA extension and adapter ligation were performed as described above for standard Illumina fragment libraries with the following exceptions: the genomic DNA (9 μg of NA12878 total human DNA) was sheared in three batches of 3 μg each; the ligation reaction contained 120 pmol pre-annealed full-length paired-end Illumina adapters [21]; the product of the adapter-ligation was size-selected to an apparent size range of 320 to 350 bp relative to a double-stranded size marker.

### qPCR

Primer pairs for 'PER' and human qPCR assays, their genome of origin, sequence, length and GC-content of amplicons are listed in Tables S1 and S2 in Additional file 2. These tables also contain their designation as either 'low', 'mid' or 'high' GC qPCR assays, indicating which qPCR protocol was used. Low and mid-GC qPCR reactions contained 5 μl Power SYBR Green PCR master mix (Applied Biosystems), 3 μl of primer pair (see Tables S1 and S2 in Additional file 2 for the recommended concentration of the primer pair), 0.6 μl of template DNA in T_10_E_0.1 _buffer (sample, standard curve or blank) and 1.4 μl H_2_O for a final volume of 10 μl. High GC qPCR reactions contained 6 μl Power SYBR Green PCR master mix (Applied Biosystems), 2.4 μl 5M betaine, 3 μl of primer pair, 0.6 μl of template DNA in T_10_E_0.1 _buffer in a final volume of 12 μl. qPCR reactions were performed on a 7900HT real-time PCR instrument (Applied Biosystems). The thermocycling protocol was 2 minutes at 50°C; 10 minutes at 95°C; 50 × (20 s at 95°C, 20 s at 47.5°C, 120 s at 55°C) for low GC assays, 2 minutes at 50°C; 10 minutes at 95°C; 50 × (20 s at 95°C, 20 s at 55°C, 40 s at 60°C) for mid-GC assays and 2 minutes at 50°C; 10 minutes at 95°C; 50 × (20 s at 95°C, 60 s at 60°C) for high GC assays. The standard curve for absolute quantification of 'PER' amplicons was prepared from the very same mixture of 'PER' DNA that was used for preparing the Illumina fragment libraries. It consisted of a five-fold dilution series ranging from 2.6 ng down to 170 fg (nominally 75,125 down to 5 haploid 'PER' genome equivalents) and a non-template control. Approximately 100 pg 'PER' sample libraries was added per reaction as determined by a Quant-iT dsDNA HS assay of the sample. The known standards for quantification of human loci were a five-fold dilution series of human female genomic DNA (Promega, Madison, WI, USA) ranging from 33 ng down to 2.1 pg per reaction. Approximately 1 ng of human fragment library was added per reaction. All reactions were performed in duplicate. Data points that were equal to or less than three-fold above background (no template) amplification were omitted. Duplicate qPCR measurements were averaged. To normalize for differences in the DNA concentration between 'PER'-derived samples, the average quantity of each amplicon in a given sample was divided by the mean average quantities of the 48% and 52% GC amplicons in the same sample. Hence, all GC-bias plots meet at 50% GC. For human-derived samples, we divided the average quantity of each amplicon by the mean quantity of four control loci (9 to 12 in Table S2 in Additional file 2).

### Isolation of biotin-adapter-ligated DNA fragments by streptavidin capture

Non-phosporylated biotinylated Illumina adapters were prepared by annealing 5'-ACACTCTTTCCCTACACGACGCTCTTCCGATCxT and 5'-GATCGGAAGAGCGGTTCAGCAGGAATGCCGAG-3BioTEG (IDT, Coralville, IA, USA) where 'x' denotes a nuclease-resistant phosphorothioate linkage and '3BioTEG' a biotin attached via a 15-atom linker at the 3' end. Ligation to end-repaired and 3'-dA extended genomic DNA fragments was carried out as described above for regular adapters. Ligations were cleaned up by Qiagen MinElute and 1× AmPure XP beads (Beckman Coulter Genomics). M-280 streptavidin Dynabeads (50 μl; Invitrogen) were washed three times and resuspended in 400 μl of 1 M NaCl, 10 mM Tris-HCl, pH 7.5, and 1 mM EDTA. After adding the ligation reactions (26 μl), the beads were kept in suspension for 30 minutes at room temperature on a rotary mixer, pulled down and washed once for 15 minutes at room temperature with 0.5 ml 1× SSC/0.1% SDS. Ligation products were eluted by resuspending the beads in 50 μl 0.1 M NaOH. After 15 minutes at room temperature, the beads were pulled down, and the supernatant (containing melted-off single-stranded genome fragments with the non-biotinylated adapter oligo attached) was transferred to a tube containing 70 μl 1 M Tris-HCl, pH 7.5. The neutralized eluate was desalted and concentrated on a Qiagen MinElute column.

### Illumina sequencing and sequence analysis

Sequencing was performed in paired-end mode with Illumina HiSeq 2000 chemistry using Illumina data analysis pipeline version 1.8. 'PER' fragment libraries were sequenced at densities of 2.9 to 3.2 million clusters per tile. Paired-end reads (median insert size 398 to 405 bp) were aligned by BWA [22] version 0.5.7-6 (r1399) to the 'PER' reference, which was constructed by concatenating the references of the component species. Each PCR condition was represented by a single lane of data consisting of 124 to 142 million mapped reads, each 101 bases in length. PCR-amplified and PCR-free human libraries were sequenced at 3.1 and 3.9 million clusters per tile, respectively. Paired-end 101-base reads (median fragment insert size 172 bp and 166 bp, respectively) were aligned by BWA to human reference sequence GRCh37/hg19. Relative representations of the qPCR amplicon loci in sequencing data were determined by locating each amplicon in the 'PER' reference genome and comparing the average number of reads per base in each amplicon to the average number of reads per base across the entire 'PER' genome (excluding ambiguous/unknown bases). Since one lane of Hi-Seq data per library provided only five- to six-fold coverage of the human genome - not sufficient to calculate a meaningful coverage statistics for any given single locus - we calculated the mean read coverage per base for all 100 loci at once and divided this number by the genome-wide average. The mean sequence coverage of 50-bp windows at a given %GC bin was the number of observed reads that aligned to the 50-bp windows divided by the number of read alignments one would expect for perfectly even coverage given the number of 50-bp windows with this %GC. To calculate the relative coverage, the sequence coverage of each category was divided by the mean coverage of 50-bp windows from 48% to 52% GC. The number of 'PER' bases covered at less than 10% of the mean coverage was similarly determined by examining the number of reads overlapping each non-ambiguous base and comparing that to the 'PER'-wide average. The 'PER' sequencing data used for this study are available at the NCBI Sequence Read Archive under study number SRP004833 [NCBI:SRX033223, NCBI:SRX033224, NCBI:SRX033225, NCBI:SRX033226]. The human sequencing data are available under study number SRP005622 [NCBI:SRX040660, NCBI:SRX040661].

## Abbreviations

bp: base pair; 'PER': pool of genomic DNA prepared from *Plasmodium falciparum*, *Escherichia coli *and *Rhodobacter sphaeroides*; qPCR: quantitative PCR.

## Competing interests

The authors declare that they have no competing interests.

## Authors' contributions

DA, WSC and MD carried out research in the lab and analyzed qPCR data. MGR, TF and DBJ analyzed sequencing data. CR and CN coordinated the research. AG conceived the project and wrote the paper.

## Supplementary Material

Additional file 1 **Supplementary Figures 1 to 6**. Click here for file

Additional file 2 **Supplementary Tables 1 to 3**. Click here for file
