# Supplementary material for: Analyzing and minimizing PCR amplification bias in Illumina sequencing libraries
Source: Genome Biol. 2011 Feb 21;12(2):R18. doi: 10.1186/gb-2011-12-2-r18 (PMC3188800; doi:10.1186/gb-2011-12-2-r18)
Supplement: Additional file 2 — Supplementary Tables 1 to 3. [file gb-2011-12-2-r18-S2.PDF]

Table S1. qPCR primers for 36 amplicons in P. falciparum (P), E. coli (E) and R.sphaeroides (R)

P E R	Primer 1	Primer 2	conc.[1] (µM)	qPCR Protocol	Amplicon	Length	%GC
P	AAAATTTTTATTCTTTATTATTTTTA	GGTAATTTTATTTTATTATTATTTTTAT	4	Low GC	AAAATTTTTATTCTTTATTATTTTTAAAAAAAACAAAA ATAAAAATAATAATAAAATAAAATTACC	66	6.1%
P	ATATATATAAACAAAACATATAATAGTAA	TATTAATATGTAAAAAAATTTAACG	4	Low GC	ATATATATAAACAAAACATATAATAGTAATATATTAAA TTAAAACGTTAAATTTTTTTACATATTAATA	69	8.7%
P	AAATAACATATAAAAAAATAATGA	TAAGGTAAAAAATGATATAAAAATA	4	Low GC	AAATAACATATAAAAAAATAATGATAATATTTTTATAT CATTTTTTACCTTA	52	9.6%
P	ATTAAGTTATTTTAATTTACCAAGC	TTAATTTTATTATTATATATTGATTTTAAGC	1	Low GC	ATTAAGTTATTTTAATTTACCAAGCTTAAAATCAATAT ATAATAATAAAATTAA	54	11.1%
P	AAATAAAAACATTAATGAGAATTACAG	AAAAAACATTAAAAATTAAACAAAAC	4	Low GC	AAATAAAAACATTAATGAGAATTACAGTAATATATATG TTTTGTTTAATTTTTAATGTTTTTT	63	12.7%
P	AAAAAATTAACGAAATTTATACAAC	TAATTTTTAGTAATTTCATTTTGG	1	Low GC	AAAAAATTAACGAAATTTATACAACATTATTCCAAAAT GAAATTACTAAAAATTA	55	14.5%
P	TTTGTTTATATAATTTTCTTTCTGTTC	AAATAAAATACAAGAATCAATTTAATG	4	Low GC	TTTGTTTATATAATTTTCTTTCTGTTCTTTCATTAAAT TGATTCTTGTATTTTATTT	57	15.8%
E	AATAATAGATTAAAATTTCTTAACG	AATCAAAGTGTTATTTGTATGATTC	1	Low GC	AATAATAGATTAAAATTTCTTAACGATTTAAGAATCAT ACAAATAACACTTTGATT	56	16.9%
P	ATTAAAATCTACATATAACATTCCATATTC	TCTTATGGATAATTATAAATTAAACTG	1	Low GC	ATTAAAATCTACATATAACATTCCATATTCCTTAATAC AGTTTAATTTATAATTATCCATAAGA	64	18.8%
P	TATTACTATTGAGATTATAATTTGTTGATG	AATTAATAATGCTAAAACAAAAACAC	1	Low GC	TATTACTATTGAGATTATAATTTGTTGATGAAGCTGGT GTTTTTGTTTTAGCATTATTAATT	62	22.6%
E	AAGGTTTTATTAAGTTTAGAAATG	TGTGCAATAAAAACCAAATG	1	Low GC	AAGGTTTTATTAAGTTTAGAAATGATAGAAAAGTTGTA CATTTGGTTTTTATTGCACA	58	24.0%
E	TCAAGTAGCGATAAAAATCTTG	TTGCGAGATAAAATGCTTC	1	Low GC	TCAAGTAGCGATAAAAATCTTGTAAGAAGTAAAGATAA TAATCTTGAAGCATTTTATCTCGCAA	64	26.0%
P	AAACAACCAAACAAATAAACATAACG	TTTTCATTATTAGAAGGATCCATAGC	1	Mid GC	AAACAACCAAACAAATAAACATAACGCTATGGATCCTT CTAATAATGAAAA	51	27.5%
E	CTTGATTACATAGTATTACGAAAGG	GGAATATTACTTCTGGTTCTCAG	1	Mid GC	CTTGATTACATAGTATTACGAAAGGATTTTACTGAGAA CCAGAAGTAATATTCC	54	31.5%
P	CGTAATTCTTCAGCAATTCTTGG	TCATATTAAATGGTGGTGGAAAG	1	Mid GC	CGTAATTCTTCAGCAATTCTTGGAGATAACTTTCCACC ACCATTTAATATGA	52	34.6%
E	GCTTACGGCTTTATATTACGG	CAAATCGCAACTTTGATCG	1	Mid GC	GCTTACGGCTTTATATTACGGGTGAAAAACTGATGAAA TTCGATCAAAGTTGCGATTTG	59	37.3%
E	CATGTGGTTCATTCGTGTC	TTTTGATGAGTTGGTTTTGG	1	Mid GC	CATGTGGTTCATTCGTGTCGCTTATACCATAAGCCAAA ACCAACTCATCAAAA	53	39.6%
E	CAAAATGGTGTTCAGTTTTATTC	ACGTTACGCAAACCATCC	1	Mid GC	CAAAATGGTGTTCAGTTTTATTCAACAAGCCCTGGCGG ATGGTTTGCGTAACGT	54	44.4%

PER	Primer 1	Primer 2	conc.[1] (μM)	qPCR Protocol	Amplicon	Length	%GC
E	CCAGATGGCACTCCAGTC	TACACCACGGTAACAGGAAC	1	Mid GC	CCAGATGGCACTCCAGTCAAACTGCATATGTTGTTCCT GTTACCGTGGTGTA	52	48.1%
E	CCGGAAATATCGCCATAGAC	GGGATTTGAAGAACTGGCAAC	1	Mid GC	CCGGAAATATCGCCATAGACTAGCGCGACATGATCGTG TCCGGTTGCCAGTTCTTCAAATCCC	63	52.4%
R	GGCAGGACGTGGCAAAAG	GCGAGCGAATACTGTAGTTGAG	1	Mid GC	GGCAGGACGTGGCAAAAGCGGCGCATCTTTGCTCAACT ACAGTATTCGCTCGC	53	56.6%
R	GCCGACATTTTCGTGCAG	AGGTCGAGACCGAGGAGTG	1	Mid GC	GCCGACATTTTCGTGCAGGGCGGCGATTTCACCGCACT CCTCGGTCTCGACCT	53	62.2%
E	CAGGCCGTCGTCGAGTAG	GGATGCTGGAGCGTAATCC	1	Mid GC	CAGGCCGTCGTCGAGTAGCGCGGCAACCACTGGATTAC GCTCCAGCATCC	50	64.0%
E	CAGGCGCTGGCGGAGGATCT	GCGCTGCTGCCCGTCTGTTT	1	Mid GC	CAGGCGCTGGCGGAGGATCTCCGGCGAAACCTGTAAAC AGACGGGCAGCAGCGC	54	66.7%
R	GCTGCCGATGGCGCTGTTC	GCCCCATGATGCGCGGATAG	2.5	High GC	GCTGCCGATGGCGCTGTTCGGCCCCGATCGCTATCCGC GCATCATGGGGC	50	70.0%
R	GATTCGGCGTGACGGACGTG	GCCGCCGCGGATGTTCTG	2.5	High GC	GATTCGGCGTGACGGACGTGCTCGACCCGGCCCAGAAC ATCCGCGGCGGC	50	72.0%
E	CACGACCCCCGCCAGCAG	GCCGCGCAGATGGGCTACCT	2.5	High GC	CACGACCCCCGCCAGCAGCCACAGCGTGCCAGGCAGGT AGCCCATCTGCGCGGC	54	74.1%
E	GCGACCAGCGGCGCGAT	CCAGCCGTGCCCGCCA	2.5	High GC	GCGACCAGCGGCGCGATGGAGCCGTTCCCCTTCCTGGC GGGCACGGCTGG	50	76.0%
R	CCCCGGCCGAGACGAAGG	GGCGGCATGGTCGCAAACC	2.5	High GC	CCCCGGCCGAGACGAAGGGGAGGGGCGCCCCGGTTTGC GACCATGCCGCC	50	78.0%
R	CCTCGGCGCGGCTCGT	TCCCACCGGCACCTCGAGAA	2.5	High GC	CCTCGGCGCGGCTCGTGGCGGCGGGGGGCCTTCTCGAG GTGCCGGTGGGA	50	80.0%
R	CATCAGGGCCGCGAGCGT	CTGGCCGAGGCGGGGCTT	2.5	High GC	CATCAGGGCCGCGAGCGTCGCCGCGTCGGCGGGAAGCC CCGCCTCGGCCAG	51	80.3%
R	GCCCGGGCCGAGATGAGC	GGACCTCGGCGCGAACG	2.5	High GC	GCCCGGGCCGAGATGAGCGCCGCCGAGCGCGAGGCCGT TCGCGCCGAGGTCC	52	80.7%
R	ACCGCCGTGGCGCAGG	GGGGCGGGCATCACGCT	2.5	High GC	ACCGCCGTGGCGCAGGCGCCCGAGCCGCAGGCGAGCGT GATGCCCGCCCC	50	82.0%
R	CTTCGCCGCGGGCACCT	CGGGGTCGAGCGGGACG	2.5	High GC	CTTCGCCGCGGGCACCTGGCAGGCGGGGGCGCCCGTCC CGCTCGACCCCG	50	84.0%
R	GCGGTGGGGGCCGCAT	CCCGCGCCCGAGCC	2.5	High GC	GCGGTGGGGGCCGCATCCGGCGCGGGCGCAGGCTCCGG GCGCGGCTCGGGCGCGGG	56	89.2%
R	GGGCCTCAGCCGGACGTG	GCCCCGCGCCTCGC	5	High GC	GGGCCTCAGCCGGACGTGCGGCCCGCGTGGCCGCCGCG GCGGCGGGCGAGGCGCGGGGC	59	89.8%

[1] Concentration of working stock of primer pair in μM

Table S2. qPCR primers for 12 human loci. Loci #1-8 are known to be under-represented in Illumina sequencing data. # 9-12 are normal controls.

#	Primer 1	Primer 2	conc.[1] (µM)	qPCR Protocol	Amplicon	Length	%GC
1	CCGCGGCGTGTTGCTATGTG	ACGCGCCCCCGCACCTAT	1	High GC	CCGCGGCGTGTTGCTATGTGCTGAGCCGAGGGGATAG GTGCGGGGGCGCGT	51	70.6%
2	CGCCCTGTTCCCGGTTCC	TCGGCCCTGGGCAGCATTTA	1	High GC	CGCCCTGTTCCCGGTTCCCGAGCCGGGCCCCGGAGGC CTTTAAATGCTGCCCAGGGCCGA	60	71.7%
3	GGCTCAGGGGCCCGGACAT	GGAGCGCGAGCCAGCAAGAG	1	High GC	GGCTCAGGGGCCCGGACATCGCGCAGGCGCCTCTTGC TGGCTCGCGCTCC	50	76.0%
4	GAGCGAGGCTGAACGCCTGA	TGGGCGAGGTTTGGCGATGT	1	High GC	GAGCGAGGCTGAACGCCTGACGTCAAGGCGACATCGC CAAACCTCGCCCA	50	64.0%
5	GCCACCACGGGACAGGAAGG	GGAGCCTGCTTGCAAAGCTGAGG	1	High GC	GCCACCACGGGACAGGAAGGTGAGATCCGGGACCTCA GCTTTGCAAGCAGGCTCC	55	63.6%
6	GGGGCTCGCCAGGTGCAG	CCACCAGCGCCCTAGCCTCT	1	High GC	GGGGCTCGCCAGGTGCAGCCGCGGCGGCCAAGAGGCT AGGGCGCTGGTGG	50	78.0%
7	GGACCCAGAGCAGGACAGCGG	TCGCTCACCTGACGAGAGGCAG	1	High GC	GGACCCAGAGCAGGACAGCGGCCCGGAGGACCTGCCT CTCGTCAGGTGAGCGA	53	69.8%
8	GGACCCGCTTGCCGCACTTAG	AGGGACTCGGGGCGCTGC	1	High GC	GGACCCGCTTGCCGCACTTAGGCGAGTCTCCCCTGCA GCGCCCCGAGTCCCT	52	71.2%
9	TGGAATCAAGGCTCAGTCCAACTGC	CAGGAATGGAATCTGTCACTGGAGGTG	1	High GC	TGGAATCAAGGCTCAGTCCAACTGCACCTCCAGTGAC AGATTCCATTCCTG	51	51.0%
10	CCTTGCGGGTGGCTCTGTGG	CCCAGTCCAGGCAGTCAGACAAGG	1	High GC	CCTTGCGGGTGGCTCTGTGGCAGCCACCTTGTCTGAC TGCCTGGACTGGG	50	66.0%
11	GGGCCAGGGGACCTGAGTGAG	TGCAAGGACTCCAAAGTGGTGAGG	1	High GC	GGGCCAGGGGACCTGAGTGAGGCTCCGTTGTTCTCAC CTCACCACTTTGGAGTCCTTGCA	60	60.0%
12	GGCAGCACGGGGCTCAGAAC	CGGCCCAAGAGGTGCCAGAAG	1	High GC	GGCAGCACGGGGCTCAGAACAGCTCCACCTCCTTCTG GCACCTCTTGGGCCG	52	67.3%

[1] Concentration of working stock of primer pair in µM

Table S3. Coordinates of 100 GC-rich test loci in the human reference genome (hg19). All loci are on or near an annotated transcription start site and known to be underrepresented in whole-human-genome sequencing reads from previous experiments.

chr17:78194099-78194299	chr18:12947883-12948083
chr19:33685499-33685699	chr9:139581811-139582011
chrX:153979248-153979448	chr11:70244512-70244712
chr16:30381422-30381622	chr14:51297739-51297939
chr20:590810-591010	chr21:43373899-43374099
chr2:238600829-238601029	chr8:146024161-146024361
chr6:117803720-117803920	chr7:127228417-127228617
chr8:48173442-48173642	chr2:85132663-85132863
chr10:108924192-108924392	chr1:1209134-1209334
chr9:94186044-94186244	chr13:51796407-51796607
chr19:45147126-45147326	chr20:60982239-60982439
chr15:57668605-57668805	chr21:35987282-35987482
chr14:94640549-94640749	chr7:105029241-105029441
chr9:36136642-36136842	chr22:38302246-38302446
chrX:48814793-48814993	chr19:11071498-11071698
chr15:26108249-26108449	chr22:39745854-39746054
chr6:146864728-146864928	chr17:2496823-2497023
chr4:99182427-99182627	chr8:141645545-141645745
chr1:156023416-156023616	chr12:120806858-120807058
chr15:68871473-68871673	chr19:1354910-1355110
chr22:44893905-44894105	chr1:41249584-41249784
chr18:21269462-21269662	chr21:33784652-33784852
chr2:47168213-47168413	chr13:99229296-99229496
chr6:43423186-43423386	chr9:140149659-140149859
chr1:9648877-9649077	chr11:76777892-76778092
chr14:105487393-105487593	chr7:156742317-156742517
chr3:126076137-126076337	chr11:75917474-75917674
chr20:31350091-31350291	chr11:67210883-67211083
chrX:7066089-7066289	chr2:180129250-180129450
chr18:8609343-8609543	chr5:53813493-53813693
chr21:44394543-44394743	chr19:42387167-42387367
chr15:60884607-60884807	chr7:143059740-143059940
chr1:149822528-149822728	chr7:141401853-141402053
chr11:70116723-70116923	chr1:225615684-225615884
chr8:143808521-143808721	chr17:66244045-66244245
chr15:78556889-78557089	chr3:196295625-196295825
chr9:136024488-136024688	chr20:388609-388809
chr5:81267744-81267944	chr20:49547427-49547627
chr4:2757961-2758161	chr11:13299225-13299425
chr1:33815399-33815599	chr20:48184607-48184807
chr2:74374939-74375139	chr5:139175306-139175506
chr17:4710321-4710521	chr9:100954822-100955022
chr20:982804-983004	chr1:53098966-53099166
chr4:170947329-170947529	chr3:38691063-38691263
chr19:55795434-55795634	chr9:115095812-115096012
chr14:24867892-24868092	chr18:44556349-44556549
chr5:139726088-139726288	chr20:62339265-62339465
chr11:75141133-75141333	chr1:236849670-236849870
chr1:1950668-1950868	chr17:80376362-80376562
chr3:126194662-126194862	chr1:65432087-65432287
