# Supplementary material for: Analyzing and minimizing PCR amplification bias in Illumina sequencing libraries
Source: Genome Biol. 2011 Feb 21;12(2):R18. doi: 10.1186/gb-2011-12-2-r18 (PMC3188800; doi:10.1186/gb-2011-12-2-r18)
Supplement: Supplementary file 2 [file gb-2011-12-2-r18-S1.DOC]

Supplementary Figures 1-6

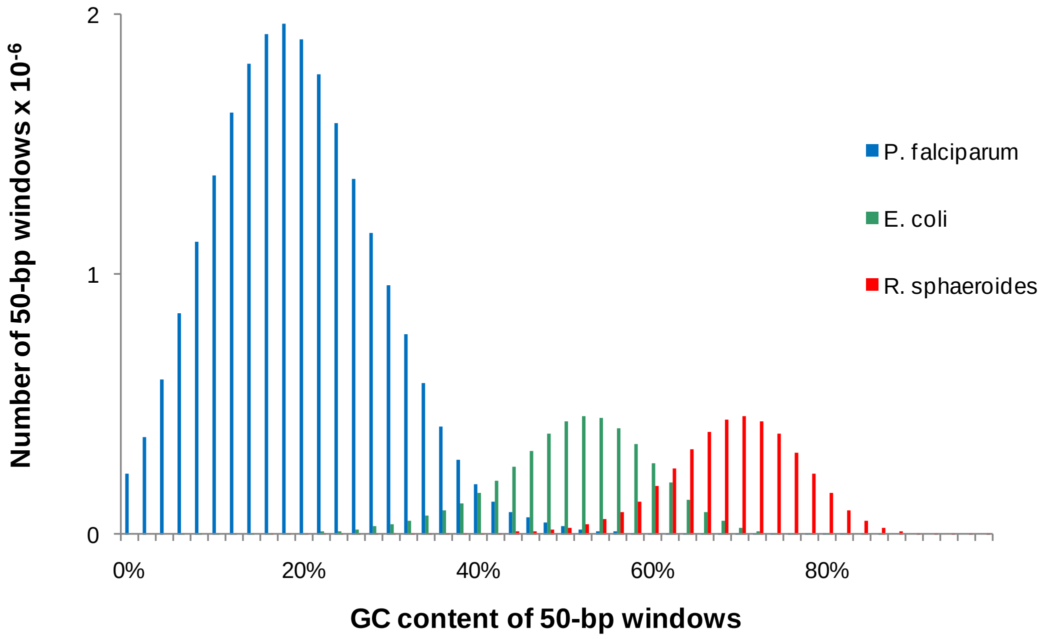

Figure S1. Base composition of the three genomes. Histograms of the percent GC in 50-bp window for the genomes of *Plasmodium falciparum*, *Escherichia coli* and *Rhodobacter sphaeroides*. Most of the DNA in the equimolar composite "PER genome" is AT-rich because the AT-rich *P. falciparum* genome (23 Mb) is 5 times larger than the GC-rich *R. sphaeroides* genome (4.6 Mb) and the intermediate *E. coli* genome (4.6 Mb).

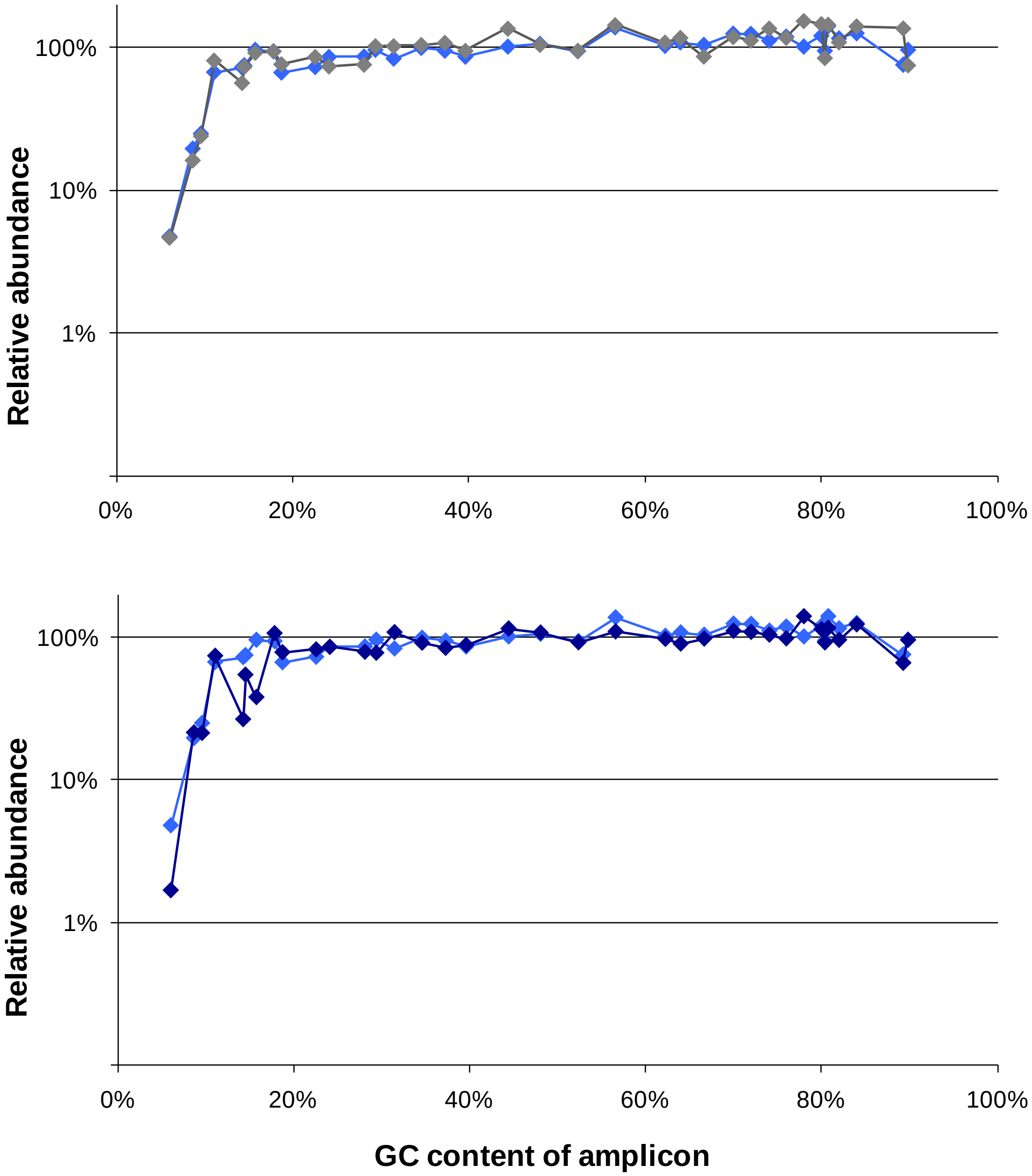

Figure S2. Testing the optimized enrichment PCR on two different thermocyclers and with long-insert fragment libraries. (a) Shown is the GC-bias profile of a ~180-bp fragment library PCR-amplified by AccuPrime Taq HiFi (long denat., primer extension at 65 °C) on the fast ramping thermocycler #1 (blue line) and on the slow ramping thermocycler #3 (gray line). (**b**) Shown are the GC-bias profiles of a short-insert (~180 bp; blue line, same as in **a**) and of a long-insert (~360 bp) Accuprime-amplified fragment library (dark blue).

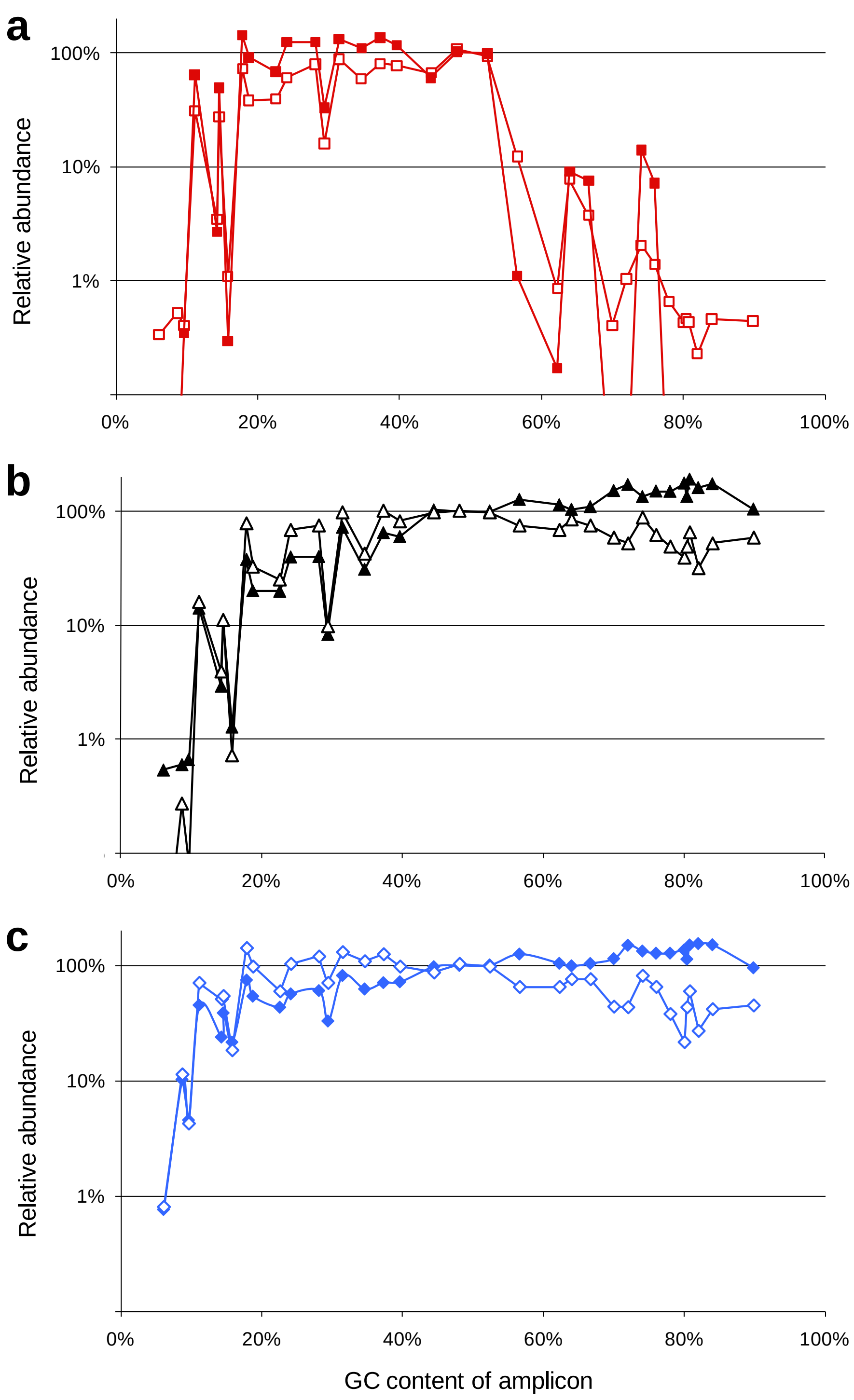

Figure S3. Comparing input library and output sequencing data. A ~400 bp fragment library was amplified with (**a**) Phusion HF (short denat, fast ramp), (**b**) Phusion HF (long denat., 2M betaine) or (**c**) Accuprime Taq HiFi (long denat., primer extension at 65˚C). Shown is the relative abundance of loci in each library as determined by qPCR (filled symbols) and the relative abundance of Illumina sequencing reads covering these loci in one lane of Hi-Seq (open symbols). Data sets were normalized to the average of the two loci closest to 50% GC.

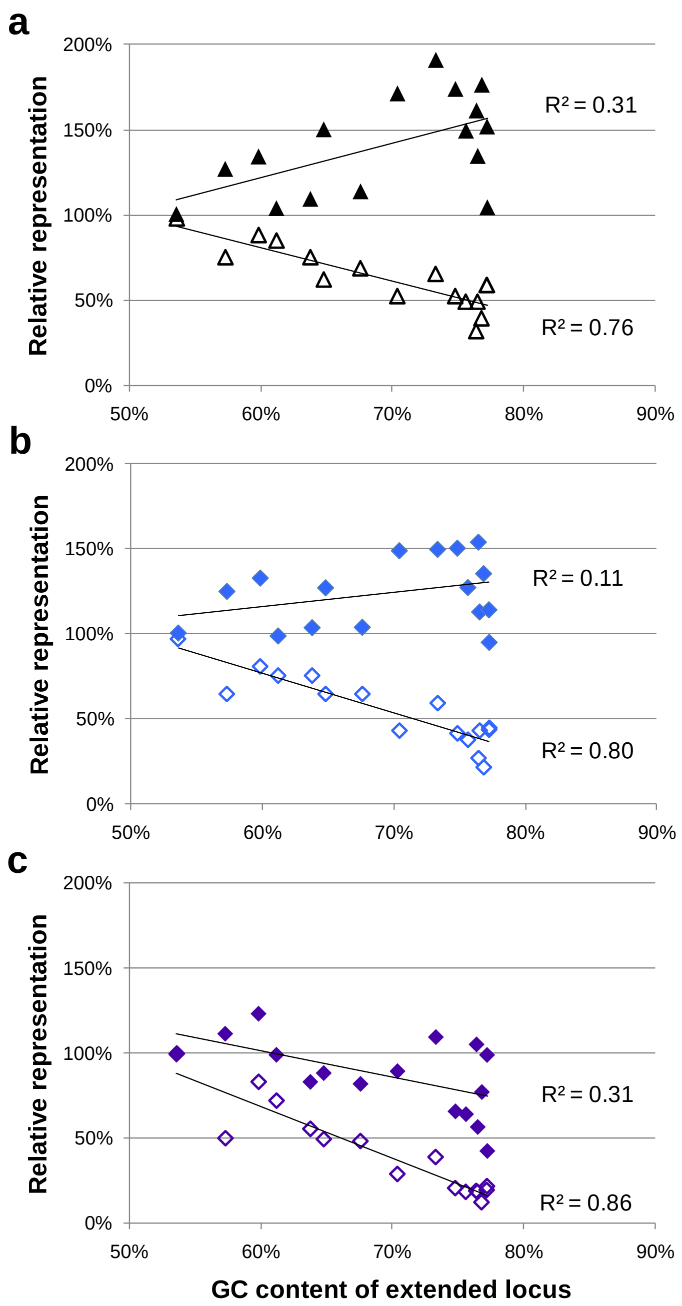

Fig. S4. Abundance of GC-rich amplicons as a function of the GC content of a ~250-bp window. Shown is the relative abundance in fragment libraries (filled symbols) and the relative sequence coverage (open symbols) of 16 qPCR amplicons ranging from 50 bp to 63 bp in length plotted over the GC content of the amplicon plus 100 bp on either side. The libraries had ~400-bp inserts and were amplified using (**a**) Phusion HF (long denat., 2M betaine), (**b**) Accuprime Taq HiFi (long denat., primer extension at 65˚C, or (**c**) Accuprime Taq HiFi (long denat., primer extension at 60˚C). The relative sequence coverage of the qPCR amplicons decreases almost linearly with GC content of the extended loci (R^2 values from 0.76 to 0.86). When plotted over the GC content of the amplicons proper, the R^2 values for sequence coverage were lower (0.50) in all three cases. Depending on the PCR conditions, the abundance of qPCR amplicons in the library can increase (**a, b**) or decrease (**c**) with the GC-content. Sequence coverage of high-GC loci is lower than their abundance in the library due to bias downstream of library construction.

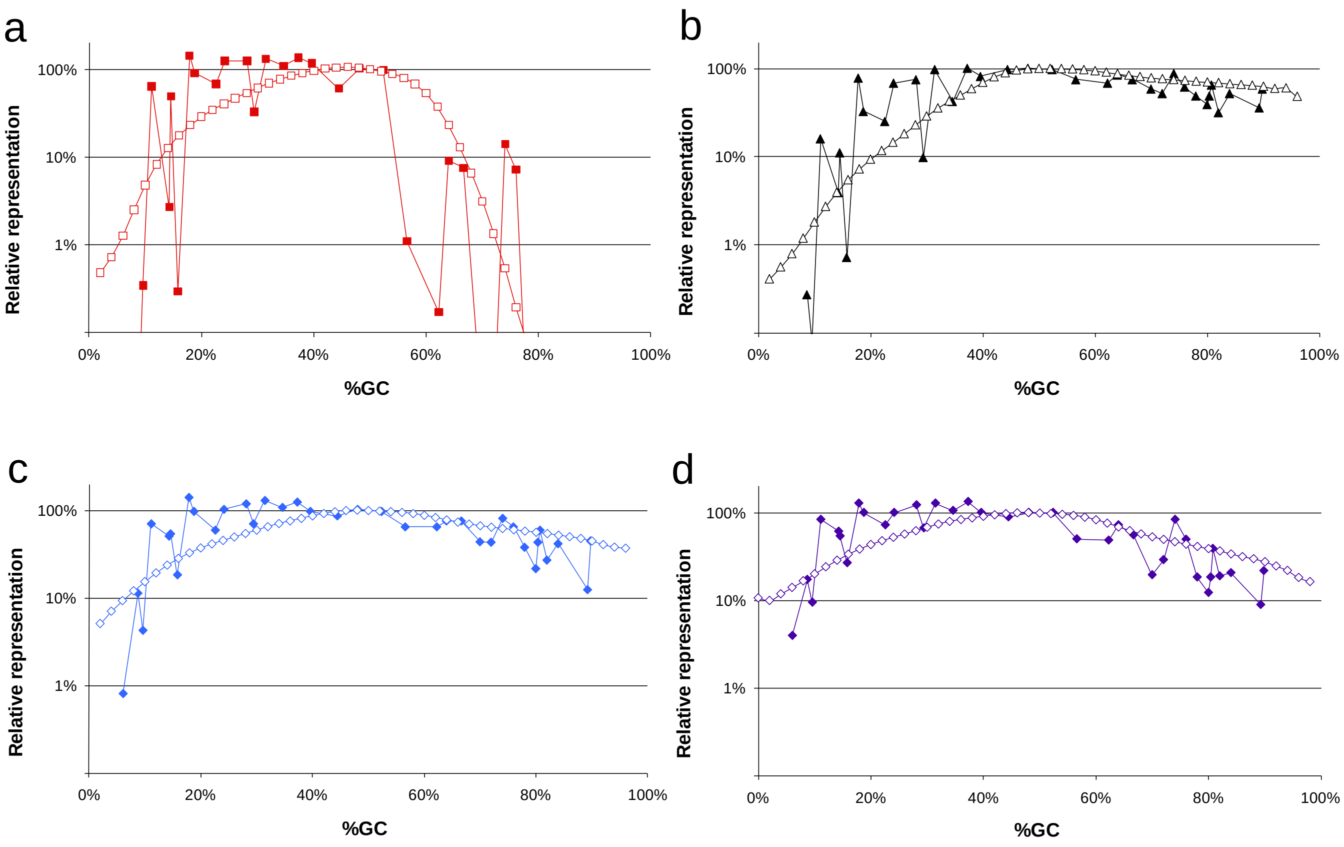

Figure S5. Comparison of qPCR data and genome-wide sequence data. The conditions for PCR-amplification of the ~400-bp fragment library were (**a**) Phusion HF (short denat., fast ramp), (**b**) Phusion HF (long denat., 2M betaine) or (**c**) Accuprime Taq HiFi (long denat., primer extension at 65˚C) and (**d**) Accuprime Taq HiFi (long denat., primer extension at 60˚C). Squiggly lines are the abundance of sequencing reads aligning to just the 36 qPCR amplicon sequences normalized to the average of the two loci closest to 50% GC. Smooth lines are the ratio of observed to expected (unbiased) average read coverages of 50-bp windows in 2% GC bins normalized to the average of the bins from 48% to 52% GC.

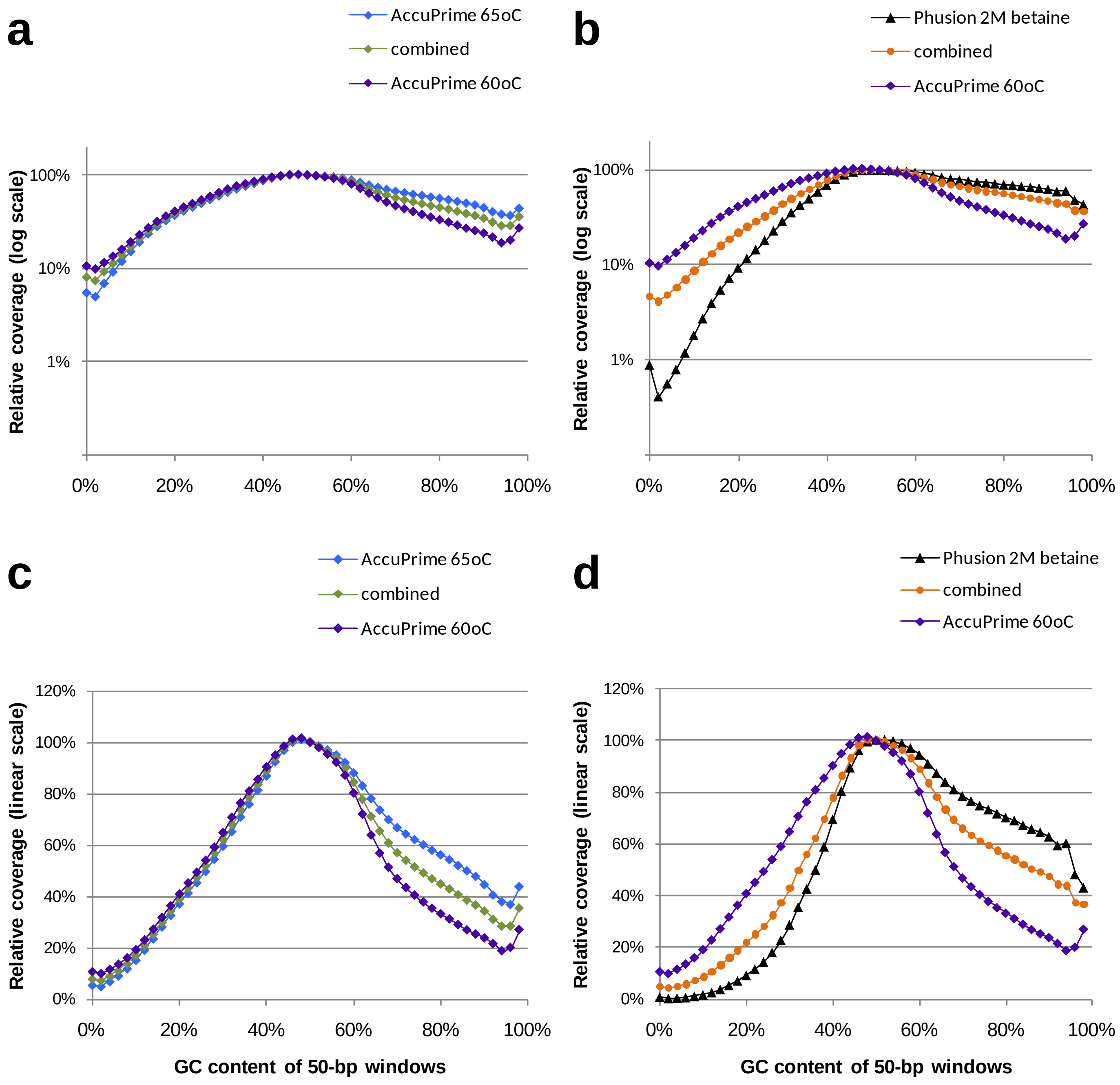

Figure S6. Pooling sequencing reads from differently amplified libraries. Shown are GC-bias curves for a single lane of Hi-Seq sequencing reads from two differently amplified libraries and composite GC-bias plots for the reads from the same two lanes combined. The ~400-bp libraries were amplified with AccuPrime Taq HiFi with primer extension at 65˚C and 60˚C (**a, c**) and Phusion long denat. with 2M betaine and AccuPrime HiFi Taq with primer extension at 60˚C (**b, d**). The y-axis is the mean coverage of 50-bp windows in the “PER” genome having the %GC indicated on the x-axis divided by the mean coverage of the mid-GC (48- 52%) references plotted on a $\log_{10}$ (**a, b**) or linear scale (**c, d**).
